# MicroRNAs Patterns as Potential Tools for Diagnostic and Prognostic Follow-Up in Cancer Survivorship

**DOI:** 10.3390/cells10082069

**Published:** 2021-08-12

**Authors:** Ilaria Conti, Carolina Simioni, Gabriele Varano, Cinzia Brenna, Eva Costanzi, Luca Maria Neri

**Affiliations:** 1 Department of Translational Medicine, University of Ferrara, 44121 Ferrara, Italy; ilaria.conti@unife.it (I.C.); gabriele.varano@unife.it (G.V.); cinzia.brenna@unife.it (C.B.); eva.costanzi@unife.it (E.C.); 2 Department of Life Sciences and Biotechnology, University of Ferrara, 44121 Ferrara, Italy; carolina.simioni@unife.it; 3 LTTA–Electron Microscopy Center, University of Ferrara, 44121 Ferrara, Italy

**Keywords:** long-cancer survivor, microRNA, biomarkers, cancer prognosis

## Abstract

Advances in screening methods and pharmacological treatments are increasing the life expectancy of cancer patients. During recent decades, the community of long-term disease-free cancer survivors (LCS) has grown exponentially, raising the issues related to cancer follow-up. Cancer relapse and other cancer-related diseases, as well as lifestyle, influence cancer survival. Recently, the regulatory role of microRNAs (miRNAs) in gene expression and their involvement in human diseases, including cancer, has been identified. Extracellular circulating miRNAs (ECmiRNAs) have been found in biological fluids and specific ECmiRNAs have been associated with cancer development and progression or with a therapy response. Here, we focus on the pivotal role of ECmiRNAs as biomarkers in cancer diagnosis and prognosis. Then, we discuss the relevance of ECmiRNAs expression in cancer survivors for the identification of specific ECmiRNAs profiles as potential tools to assess cancer outcome and to control LCS follow-up.

## 1. Introduction

The International Agency for Research on Cancer (IARC) recently described cancer as a globally spreading disease involving 20% of the worldwide population and causing high mortality, ranging from 12% in men to 9% in women [1]. Cancer is identified as the second leading cause of death, but improvements in early tumor diagnosis combined with new therapeutic treatments are boosting patient survival [2]. Since 2015 in the European Union, total cancer mortality rates have resulted in a 5-year mortality decline of about 5% in men and 4% in women [3].

Cancer survivors or long-term disease-free cancer survivors (LCS) are a growing community of cancer patients with a life expectancy of several years after the diagnosis. According to the IARC evaluation of December 2020, more than 50 million people worldwide are alive 5 years after cancer is diagnosed [4]. The survival rate after cancer diagnosis is not only related to cancer recurrence and to a healthy lifestyle, but also to the development of diseases deriving from cancer treatments. Therefore, risk factors associated with the neoplastic diseases influence cancer survival [5,6], as described in a recent study showing an increased risk of cardiovascular diseases in survivors of different cancer types [7].

MicroRNAs (miRNAs) have been identified as biomarkers for human disorders, including cancer [8]. These 18–23 nucleotides-long RNA sequences regulate cellular gene expression influencing several biological processes, such as cell proliferation, mitochondrial functions and organ development [9,10]. After annealing to complementary mRNA targets, miRNAs interfere with mRNAs translation or promote their degradation [11]. Since their regulatory role, alterations of intracellular miRNAs have been associated to several diseases other than cancers, including cardiovascular illnesses [12]. Moreover, miRNAs have been identified extracellularly in many biological fluids (i.e., blood, urine, tears) [13,14]. The association of extracellular circulating miRNAs (ECmiRNAs) with extracellular vesicles (EVs), high density lipoproteins (HDLs) and proteins, protects them from degradation and ensures the ECmiRNAs stability in RNAse-rich biological fluids, such as blood [15]. Stable ECmiRNAs act as mediators in cell-to-cell communication, modulating the biological processes of neighboring cells by gene expression regulation, such as the establishment of a favorable niche around the primary tumor, within the tumor microenvironment [16]. ECmiRNAs expression has been suggested as a biomarker in pathological conditions: for example, decreased levels of miR-17-5p and miR-20a were observed in the plasma of breast cancer patients with cardiotoxicity in comparison to cancer patients without cardiotoxicity, arising a potential diagnostic role in cardiovascular diseases [17].

miRNAs can be potential diagnostic and prognostic tools to improve cancer outcome. Investigations on novel highly informative and specific biological molecules (ECmiRNAs) can lead to early diagnosis of cancer recurrence and improve cancer risk stratification in LCS [18]. Moreover, ECmiRNAs can give feedback to adopt the most appropriate pharmacological treatment and to assess therapy response in order to achieve a precision drug therapy and, therefore, to enhance cancer survival.

## 2. miRNAs’ Characteristics and Functions

### 2.1. miRNAs Biogenesis and Gene Regulation

miRNAs are small noncoding RNAs involved in the regulation of gene expression interacting with the target mRNAs [19]. Starting from a longer nucleotide sequence (several hundreds of nucleotides) named primary miRNA (pri-miRNA), subsequent processes lead to a mature single strand miRNA capable of regulating biological functions [19]. In the nucleus, the pri-miRNAs are transcribed mostly by nonprotein coding genes (about 50%) and intragenic regions, mainly introns, while relatively few transcripts result from non-coding exons [20]. The generated pri-miRNA has a characteristic hairpin structure which is recognized and processed by the microprocessor complex [21]. The double-stranded RNA (dsRNA) binding protein DGCR8 (DGR8 microprocessor complex subunit, also named Pasha) and the RNAse III enzyme DROSHA (drosha ribonuclease III), components of the microprocessor complex, cleave the pri-miRNA, generating a 70–120 nucleotides-long precursor miRNA (pre-miRNA) with an overhang of two nucleotides at the 3′ end [22]. Within the pre-miRNA duplex, the two miRNAs strands are differently named as 5p or 3p in accordance with their direction: 5p- and 3p- strands originate from the 5′ and the 3′ ends, respectively [23]. The resulting pre-miRNA is translocated by the nuclear transport receptor protein Exportin 5 (XPO5)/GTP-binding nuclear protein Ran (RanGTP) complex into the cytoplasm, where the final steps of the canonical pathway of miRNA biogenesis can occur [24]. In the cytoplasm, the terminal loop of the pre-miRNA is removed by the ribonuclease III Dicer1 (DICER1) together with the kinase EIF2AK2 (eukaryotic translation initiation factor 2 alpha kinase 2) and the dsRNA-binding proteins TARBP and PRKRA (TAR (HIV-1) RNA binding protein and protein activator of interferon induced protein kinase EIF2AK2, respectively), obtaining a 18–23 nucleotide-long mature miRNA duplex [25]. On the other hand, noncanonical miRNAs biogenesis occurs independently from DROSHA/DGCR8 and DICER1 cleavages [26]. At the end of both canonical and noncanonical miRNAs biogenesis pathways, mature single strand miRNAs regulating the gene expression are generated.

Within the mature miRNA duplex, the two strands are distinguished as a guide strand or passenger strand in accordance with their association, or lack of, with Argonaute RISC complex (AGO) proteins in the assembly of an RNA-induced silencing complex loading complex (RISC) [27]. Generally, the guide strand is loaded into the RISC-loading complex, while the passenger strand is usually degraded [28]. However, the degradation of the passenger strand does not always occur, and both the two mature single strand miRNAs can be loaded into AGO proteins regulating gene expression. For example, miR-574-5p and miR-574-3p act oppositely in gastric cancer: a 5p-strand promotes cancer progression, while a 3p-strand suppresses it and is therefore related to a better cancer prognosis [29].

Once the mature miRNA guide strand is loaded into an AGO protein (AGO1-4 in humans) [30], the gene expression regulation can occur in presence of both the other RISC-loading complex components and the mRNA target [31,32]. miRNAs anneal to a complementary sequence on the 5′ or 3′ UTR of their mRNA targets, named miRNA Response Element (MRE) [33]. The degree of their engagement influences the mRNA fate, which can be degraded or its translation can be repressed [34,35].

Evidences showed miRNAs as pleiotropic and redundant factors in the regulation of biological processes. Indeed, a single mRNA may be targeted by multiple miRNAs, and one miRNA can bind to different mRNAs [36].

### 2.2. Extracellular miRNAs as Cell Messengers

Several studies observed the presence of several various miRNAs in extracellular fluids such as blood, urine and cell culture medium [15]. The increasing number of studies analysing extracellular circulating miRNAs (ECmiRNAs) suggest the existence of systems preserving miRNAs from their potential extracellular degradation. Plasma ECmiRNAs are remarkably stable when stored at room temperature, subjected to different pH or multiple freeze–thaw cycles, in comparison to synthetic miRNAs and mRNAs [37,38]. The encapsulation and transport of ECmiRNAs by extracellular vesicles was first hypothesized as a protecting mechanism [39,40]. Moreover, two different contemporary studies observed ECmiRNAs from plasma or cell culture medium mainly associated to AGO proteins (90%) [41,42]. Currently, circulating ECmiRNAs can be found within extracellular vesicles (EVs) or bound to proteins. Exosomes, microvesicles and apoptotic bodies are the main EVs enwrapping ECmiRNAs [43,44]. Differences in dimension, composition and biosynthesis distinguish the various EVs [45]: (i) exosomes are 30–100 nm in diameter vesicles that originate by internal budding of early endosomes [46]; (ii) microvesicles are both heterogeneous in size (i.e., from 100 to 1000 nm in diameter) and composition, since they originate by outward budding or fission of plasma membrane [47]; (iii) apoptotic bodies are larger vesicles of 1–5 µm in diameter released by dying cells [48]. Among the non-packaged ECmiRNAs, AGO2 and HDL protect them from the extracellular degradation [42,49]. Circulating miRNAs identified in cerebrospinal fluid were mostly immunoprecipitated by anti-AGO2 antibodies, suggesting their predominant association with AGO proteins [50]. A potential diagnostic role of this complex was hypothesized due to the variation of AGO2/ECmiRNAs concentration under pathological conditions, such as colorectal cancer [50].

The stability of ECmiRNAs in the extracellular fluids proposes the existence of their key roles in gene expression regulation among cells, rather than resulting as byproducts of cellular activities [19]. Furthermore, the release of ECmiRNAs is a controlled process. Ceramide is a sphingolipid on plasma membrane involved in the secretion of exosomal miRNAs. Inhibition of the sphingomyelin phosphodiesterase 2 (SMPD2) enzyme in HEK293 cells caused reduced ceramide biosynthesis, resulting in decreased exosomal miRNAs [51]. Recently, the GGAG and GGUC conserved sequences (EXO and extra-seed EXO [hEXO] sequence, respectively) at the 3′ end of exosomal miRNAs have been identified and related to the ECmiRNAs secretion mechanism [52,53]. Evidence showed the interaction among the recognized miRNAs motif and specific cellular proteins. The sumoylated form of HNRNPA2B1 (heterogeneous nuclear ribonucleoprotein A2/B1) recognized the GGAG motif on miR-198 but not the mutated sequence in T cells [54], while the GGUC sequence was bound by the SYNCRIP protein (synaptotagmin-binding cytoplasmic RNA-interacting protein) in hepatocytes [55].

As a consequence of the intracellular concentrations of both miRNAs and their targets, a passive cellular mechanism for ECmiRNAs release has been hypothesized [56]. According to this thesis, miRNAs containing vesicles are secreted when miRNAs concentration raises at a higher level than its targeted mRNA, in order to maintain a stable intracellular miRNA:mRNA ratio [57].

## 3. miRNAs in Cancer

Early and specific predictors of tumor development and progression are key elements to achieve constant monitoring in LCS patients. miRNAs have been identified as good potential screening markers for different cancer types and, therefore, for related drug treatments [58]. Their eligibility also resides in noninvasive and nonharmful tests (i.e., blood draws), that can be conceivably repeated limitlessly.

### 3.1. Aspects of Intracellular miRNAs

miRNAs regulate at least 30% of all human genes influencing most of the biological processes, including cell fate differentiation, proliferation and host-viral infection [59]. Alteration in miRNAs biogenesis, as well as in miRNAs expression levels and functions, have been related to several human cancers [60]. Decreased human HCT-116 cell (human colorectal carcinoma cells) proliferation was observed as a consequence of the knockout of DROSHA and DICER1. Western blot and sequencing analyses showed miRNAs expression reductions of 96.5 and 96% in the absence of DROSHA and DICER1, respectively [61].

The quick malignant cellular proliferation and expansion requires continuous availability of intermediates and metabolites. Cancer cells are capable of switching their miRNA expression in order to support their metabolic activities, forcing tumor cells to perform anaerobic glycolysis instead of aerobic respiration (Warburg effect) [62]. Different glycolytic enzymes are targeted by the miR-200 family: miR-122; miR-17/92 cluster; miR-15a/16-1; miR-29; miR-326 and miR-133 to support cancer cell metabolic needs, proliferation and metastasis [63]. The PGI/AMF is one of the first glycolytic enzymes, which also induces the expression of ZEB1/ZEB2 (zinc finger E-Box-binding homeobox 1/zinc finger E-Box-binding homeobox 2), thus enhancing the epithelial-to-mesenchymal transition (EMT). In MDA-MB-231 breast cancer cells, the repression of miR-200 family (miR-200a, miR-200b, and miR-200c), as a consequence of PGI/AMF (phosphoglucose isomerase/autocrine motility factor) overexpression, resulted in increased metastasis [64].

According to their role within cancer development and progression, miRNAs can be recognized as tumor-promoting miRNAs (oncomiRs), metastasis-promoting miRNAs (metastamiRs) and tumor-suppressor miRNAs [65]. The tumorigenic role of miRNAs is also influenced by the cellular context, since they can act either as tumor suppressors or as oncomiRs [66].

The PIK3/AKT/MTOR (phosphatidylinositol-4,5-bisphosphate 3-kinase/AKT serine-threonine kinase/mechanistic target of rapamycin kinase) is one of the most activated cell signaling routes in cancer [67]. Loss of function mutations of phosphatase and tensin homolog (PTEN), a negative regulator of PIK3/AKT/MTOR [68], increase PIK3/AKT/MTOR pathway activation and are associated with cancer development [69]. Gene expression analyses by RNA-seq and miRNAs microarray in biopsy or surgery tissue from 217 colorectal carcinoma patients observed the association between miRNAs and genes involving a PIK3/AKT signaling pathway, such as miR-590-5p, miR-106b and miR-93 acting as PTEN suppressors [70]. In SUM149 breast cancer cells, miR-181c inhibited the PTEN protein expression, targeting its 3′UTR mRNA, and promoted breast cancer proliferation [71]. Upregulation of miR-181a (a member of the miR-181 cluster) in Jurkat T-ALL cells (T-acute lymphoblastic leukaemia) reduced EGR1 (early growth response one) level, inducing G1/S cell-cycle progression and cell proliferation [72].

The same miRNAs may have different targets within diverse cancer types. MiR-105 was upregulated in triple-negative breast cancer tissue promoting chemoresistance, stemness and metastasis, acting on a WNT/β-catenin pathway [73]. In hepatocellular carcinoma, miR-105 downregulation activated the PIK3/AKT signaling, enhancing cancer proliferation both in vitro and in vivo [74].

Specific groups of miRNAs (clusters), and their relative expression levels may disclose a set of information with a diagnostic and prognostic role [12,16]. The first evidence of miRNAs’ involvement in human cancer dates back to 2002, when miR-15a and miR-16-1 dysregulation was associated to B-cell chronic lymphocytic leukemia (B-CLL) [75]. The 13q14 chromosome deletion, present in over 50% of B-CLL cases, induces the loss of the tumor suppressor miR-15a/miR-16-1 cluster (miR-15/16). Downregulation of these miRNAs promotes overexpression of several oncogenes including BCL2 (BCL2 apoptosis regulator), inhibiting apoptosis of tumor cells and enhancing cell proliferation [76,77,78]. Moreover, deletions or loss of functions (mutations) of the miR-15/16 were reported in a variety of tumors other than CLL, such as melanoma, colorectal and prostate cancer [79].

### 3.2. Extracellular miRNAs in Cancer

Recent studies have highlighted the functional role of ECmiRNAs, that after secretion are delivered towards target cells where they can induce regulatory effects. Therefore, since their role in cell-to-cell communication, ECmiRNAs have been identified as biomarkers for several human pathologies, including cancer.

The involvement of ECmiRNAs in tumorigenesis was hypothesized as a consequence of the higher number of released exosomes by cancer cells compared to normal controls [80]. In 24 h, the B42 breast cancer cell line released approximately 53 × 10^8^ exosomes per million cells, whereas nonpathological human mammary epithelial cells (HMEC B42) released 5 × 10^8^ exosomes per million cells [81].

Exosomes derived from EMT-transformed HCT-116 cells and containing miR-128-3p downregulated FOXO4 (forkhead box O4) and activated TGF-β/SMAD (transforming growth factor- β/SMAD family) and JAK (Janus kinase)/STAT3 signaling pathways in normal HCT-116 cells, inducing the EMT process [82]. The level of six ECmiRNAs (miR-19b-3p; miR-21-5p; miR-221-3p; miR-409-3p; miR-425-5p and miR-584-5p) were increased in the plasma of lung adenocarcinoma (LA) patients in comparison to healthy subjects, suggesting their potential role for LA diagnosis [83].

Colorectal cancer growth was also related to the regulation of the immune system through miR-222-EVs secretion. Decreased release of miR-222 microvesicles from implanted SW480 human colon cancer cells in mice resulted in reduced colorectal cancer growth and higher CD3+ cells infiltration of cancer tissue, suggesting a reduced immune escape [84].

Further studies investigated the messenger role of ECmiRNAs in cell-to-cell communication, highlighting their relevance within the tumor microenvironment [16,85]. Exosomal miR-21, secreted by human bronchial epithelial cancer cells (HBE), increased VEGF (vascular endothelial growth factor) in human umbilical vein endothelial cells (HUVEC), promoting angiogenesis [86]. Exosomes containing miR-105 can be released by metastatic breast cancer cells to destroy the vascular endothelium barrier targeting the tight junction protein 1 (TJP1) in endothelial cells (recipient cells) and promoting cancer metastasis [87].

Cancer therapy induces changes in the ECmiRNAs pattern of tumor cells and can become a readout of treatment efficacy. Analysis of blood samples from 68 metastatic colorectal cancer patients showed a decreased miR-126 level in the combined treatment of chemotherapy and bevacizumab [88]. Higher miR-128b level in peripheral blood was related to good prednisolone response and prognosis in children with ALL [89].

Summary of the described miRNAs involved in cancer metabolic pathways and tumorigenic processes are listed in Table 1 and Table 2.

## 4. Cancer Survival

When it was defined in 1985, the term “cancer survivor” referred to a cancer patient who went from a phase of “acute survivorship” (i.e., cancer diagnosis and primary treatments) to “permanent survivorship” (i.e., cancer remission or cancer as a chronic disease), experiencing a phase of “extended survivorship” (i.e., physical and psychological consequences following cancer treatment) [90]. Then, the American National Coalition of Cancer Survivorship (NCCS) identified the LCS as a person other than a patient, recognizing his/her needs (e.g., psychological, legal, medical) from the moment of the cancer diagnosis through the balance of life [91]. Moreover, the National Cancer Survivorship Initiative in the UK includes “patients undergoing primary treatments, in remission, with active or advanced disease and cured” in the LCS concept [92]. In Italy, survivors are generally defined as patients free of cancer disease and therapy for at least 5 years [93]. Within the LCS group, some researchers describe as “cured” survivors, the cancer survivors with a death rate comparable to general populations [94,95]. It should be taken into account that some patients reject the term “survivor” judging it as offensive [96]. Therefore, actually LCS do not correspond to a unique definition [97].

Overcoming the definition issue, the percentage of LCS among cancer patients is increasing due to early diagnosis and new advanced therapies. According to recent research, more than 68% of approximately 17 million of American LCS had a cancer history of 5 years or more, and 18% (i.e., more than 3 million) were 20-year survivors in 2019 [98]. The same research has foreseen an increase in LCS numbers to more than 22.1 million by 2030 [98].

Several factors affect the cancer survivorship, such as type and stage of cancer at diagnosis, cancer therapy and lifestyle. It is also known that cancer treatments, such as chemotherapy, lead to several side effects due to their nonspecific action targeting both cancer and normal cells [99]. Other than an increased risk of mortality, cancer therapy may worsen the quality of life [100]. For example, higher incidences of obesity, diabetes or fractures associated with osteoporosis were observed in prostate cancer survivors treated with androgen deprivation therapy (ADT) [101].

**Figure 1:**
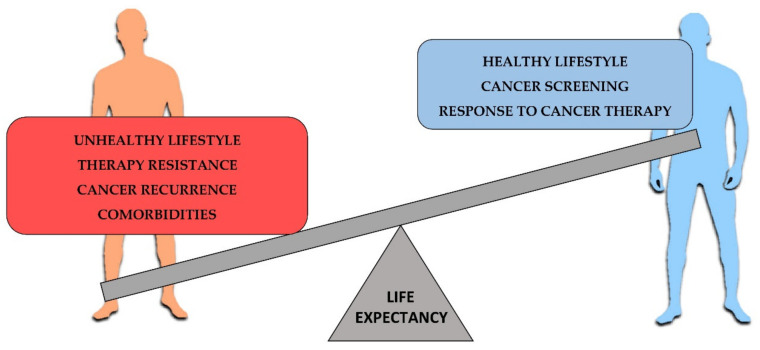
Factors affecting the cancer life expectancy. On the left, most relevant negative factors for cancer survival. On the right, positive factors contributing to increase the cancer life expectancy.

Hence, LCS life expectancy is a multifactorial condition in which patient care and lifestyle contribute to survival regardless of the tumor characteristics (Figure 1) [102,103].

## 5. miRNAs as Biomarkers in Cancer Survivors

Other than advances in pharmacological treatments, an earlier cancer diagnosis, accompanied by the most appropriate therapy, can improve the life expectancy of cancer patients [104]. Several studies showed the high sensitivity and specificity of miRNAs in cancer diagnosis [105], highlighting their potential in reducing tumor mortality as a consequence of earlier cancer detection. Recent studies evaluated the ECmiRNAs expression and correlated it to the cancer prognosis, focusing on long-term survival.

### 5.1. miRNAs for Cancer Follow-Up and Monitoring of the Therapy

Since ECmiRNAs profiles have been recognized as representative of miRNAs patterns expressed by different tumors and are capable of distinguishing their different development stages, they may act as a useful tool to predict cancer relapse or progression. Among ECmiRNAs profiles, the analysis of more than 2000 serum samples of patients with or without lung cancer, miR-1268b and miR-6075 showed 99% of both sensitivity and specificity in the detection of lung cancer [106]. Distinct ECmiRNAs patterns were detected among hepatocellular carcinoma patients and noncancerous subjects during disease progression [107].

For example, in chronic hepatitis B patients, upregulated miR-122-5p, miR-125b-5p, miR-885-5p, miR-100-5p and miR-148a-3p were found, while miR-34a-5p was identified as a biomarker for cirrhosis. Finally, downregulation of miR-424-5p and miR-101-3p and upregulation of miR-128, miR-139-5p, miR-382-5p and miR-410 were associated with larger HCC (hepatocellular carcinoma) tumor size and invasion, as well as a worse survival outcome [107].

Evaluation of ECmiRNAs patterns in breast cancer patients with more than 15 years of follow-up displayed a cancer survival potentially influenced by the expression of the five most relevant miRNAs (miR-29c, miR-143-3p, miR-187-3p, miR-205-5p and miR-210). In particular, decreased levels of both miR-210 and miR-29c were associated with a 10-year life expectancy, suggesting a better cancer prognosis [108].

Specific miRNAs patterns have been related to therapy response, thus contributing to assess its efficacy and therefore, cancer prognosis. The signature of four serum ECmiRNAs (miR-940, miR-451a, miR-16-5p and miR-17-3p) was related to the trastuzumab treatment response in 386 ERBB2+ (erb-b2 receptor tyrosine kinase 2-positive)-metastatic breast cancer patients, compared to 179 chemotherapy-treated patients. The increased expression of these four miRNAs was associated with trastuzumab resistance [109]. Analysis of miRNAs in the serum of 97 castration-resistant prostate cancer showed high levels of miR-200b, miR-429, miR-200a, miR-21, miR-200c, miR-375, miR-132, miR-20a and low levels of miR-590-5p in patients with shorter survival. Moreover, after cancer therapy with docetaxel, decreased or unchanged levels of miR-20a, miR-222, miR-20b, miR-132 or miR-25 were associated with poor prognosis. Therefore, these miRNAs patterns were suggested as potential biomarkers for docetaxel resistance and poor prognosis in prostate cancer patients [110].

Diet contributes to cancer survival and influences the ECmiRNAs pattern. In a very recent study, 42 EVs-miRNAs (among the 798 studied) were differently expressed in the plasma of overweight breast cancer survivors treated with the Mediterranean diet for eight weeks: 36 miRNAs were upregulated and six miRNAs were downregulated. The identified miRNAs were associated with different cellular pathways involved in breast cancer progression (e.g., miR-122-5p and let-7a-5p, engaged in tumor metastasis), energy and glucose metabolisms (e.g., miR-329-3p and miR-216-5p, altered in obesity), thus suggesting a positive influence of the diet [111].

ECmiRNAs have been also associated with cancer relapse prediction. qRT-PCR analysis showed a higher expression of miR-194 and miR-146-3p in the serum of patients with prostate cancer recurrence compared to nonrecurrent patients, following radical prostatectomy [112]. In another study, increased circulating miR-17, miR-20a, miR-20b and miR-106a (all members of the oncogenic cluster of the miR-17 family) in blood samples predicted a high risk of prostate cancer relapse [113]. In biliary tract cancer, the relapse after radical surgery was disclosed by six specific ECmiRNAs (miR-1225-3p, miR-1234-3p, miR-1260b and miR-1470, miR-6834-3p and miR-6875-5p). Upregulation of miR-1225-3p, miR-1234-3p, miR-1260b and miR-1470 and downregulation of miR-6834-3p and miR-6875-5p were observed in the serum samples of patients with cancer relapse. The best prognostic information with 84.6% of sensitivity, 100% of specificity and 90.0% of accuracy, was obtained using a set of three randomly selected miRNAs among the six analysed [114].

### 5.2. miRNAs to Monitor Cancer Therapy Side Effects

Other than ECmiRNAs patterns related to cancer follow-up, circulating miRNAs have been also associated with cancer therapy side effects, such as cardiotoxicity, that influence patients’ life expectancy.

At least 50% of cancer chemotherapy drugs, including doxorubicin, anthracycline and cisplatin, are known to induce oxidative stress [115]. Oxidative stress condition changes EVs and ECmiRNAs release to mediate cell–cell communication in order to counteract or alternatively boost the inflammation [116,117]. MiR-1, miR-133b, miR-146a, miR-208a, miR-208b and miR-423-5p (cardiac functions related miRNAs) were observed in plasma of 56 doxorubicin-treated breast cancer patients after each cycle of treatment. Among them, miR-1 was recognized as the best biomarker for doxorubicin-induced cardiotoxicity showing its significant increased expression in comparison to the other miRNAs, and driving a better evaluation of cardiac injuries rather than the cardiac troponin I (cTnI), in patients with cardiotoxicity [118,119]. Upregulation of miR-126-3p, miR-199a-3p, miR-423-5p and miR-34a-5p (markers of congestive heart failure [CHF]) was detected in the plasma of primary breast cancer patients treated with anthracycline-based chemotherapy that developed CHF, suggesting an impairment of heart functions as consequence of cancer therapy [120].

ECmiRNAs identified as biomarkers for cancer follow-up are listed in Table 3.

## 6. Conclusions

Changes in miRNA expression levels alter biological processes, such as cellular proliferation and metabolism. Evaluation of ECmiRNAs in biological fluids, such as blood or urine, could represent a potential routine test, based on miRNAs quick analysis, noninvasive and nonhazardous characteristics for the patients. Therefore, ECmiRNAs analysis appears conceivably endlessly repeatable, with the possibility of multiple samplings over time that are important especially in the follow-up [121]. Furthermore, since specific circulating miRNAs should reflect the miRNAs profiles within the parental tumor [53], they might provide information on the tumor stages and its modifications more rapidly than other techniques. Currently, some cancers, such as lung cancer, have low life expectancies due to a lack of distinctive symptoms that causes a late diagnosis [98]. A specific ECmiRNAs profile could be informative for cancer diagnosis and follow-up, but could also suggest the appropriate treatment to block tumor growth and to monitor therapy side effects. Recent studies evaluated miRNAs themselves as a potential cancer therapy. For example, TG221 transgenic mice treated with miR-199a-3p mimic displayed a reduction in liver cancer size and a fewer number of nodules compared to control animals [122].

It should be taken into account that ECmiRNAs expression changes as a consequence of personal characteristics, such as lifestyle (i.e., diet and physical activity) [123,124]. Limitations of ECmiRNAs analyses may be related to availability of clinical samples and amount of miRNAs content, RNA extraction methods, miRNA database errors and unstandardized statistical analyses [125,126].

Identification of biomarkers in cancer survivors is a very innovative research topic and ECmiRNAs are gaining an emerging role within it. The identification of ECmiRNAs patterns, related to specific cancer stages and progression phases, could allow us to prevent cancer relapse and to monitor therapy effects.

Knowledge of cancer characteristics, as described by ECmiRNAs, may orient toward the best individual treatment, fitting within the idea of a personalized precision medicine.

## Figures and Tables

**Table 1:** Examples of representative miRNAs involved in cancer cell lines development, progression and therapy response.

miRNA	Intracellular/Extracellular	Cells	miRNA Level	Target	Effect	Ref.
miR-15a/miR-16-1	Intracellular	B-CLL cells	↓	BCL2, MCL1, CCND1, Wnt3A	Cancer cell proliferation and inhibition of apoptosis	[78]
miR-21	Extracellular	HBE cells	↑	VEGF	Promotion of angiogenesis in HUVEC cells	[86]
miR-105	Extracellular	Metastatic breast cancer cells	↑	TJP1	Promotion of metastasis	[87]
miR-126	Extracellular	Blood sample	↓	-	Good metastatic colorectal cancer therapy response (chemotherapy and bevacizumab combination)	[88]
miR-128-3p	Extracellular	HCT-116	↑	FOXO4	Promotion of EMT	[82]
miR-181a1	Intracellular	Jurkat cells	↑	EGR1	G1/S cell-cycle progression, cell proliferation	[72]
miR-181c	Intracellular	Hepatocytes	↓	PIK3/AKT	Cancer cell proliferation	[74]
miR-200a, miR-200b, miR-200c	Intracellular	MDA-MB-231 cells	↓	PGI/AMF	Promotion of metastasis	[64]
miR-222	Extracellular	SW480 cells	↓	ATF3	Inhibition of colorectal cancer growth and promotion of immune system response	[84]

↑ Upregulation; ↓ downregulation; ATF3: activating transcription factor 3; B-CLL: B-chronic lymphocytic leukemia; BCL2: BCL2 apoptosis regulator; CCND1: cyclin D1; EGR1: early growth response 1; EMT: epithelial-to-mesenchymal transition; FOXO4: forkhead box O4; HBE: human bronchial epithelial cells; HCT-116: human colorectal cells; HUVEC: human umbilical vein endothelial cells; MCL1: MCL1 apoptosis regulator; PIK3/AKT: phosphatidylinositol-4,5-bisphosphate 3-kinase/AKT serine-threonine kinase; WNT3A: Wnt family member 3A; PGI/AMF: phosphoglucose isomerase/autocrine motility factor; TJP1: tight junction protein 1; VEGF: vascular endothelial growth factor.

**Table 2:** Examples of representative miRNAs from samples of cancer patients involved in tumor development, progression and therapy response.

miRNA	Intracellular/Extracellular	Tissue	miRNA Level	Target	Effect	Ref.
miR-93, miR-106, miR-590-5p,	Intracellular	Colorectal carcinoma biopsy	↓	PTEN	Activation of PIK3/AKT pathway; cancer development	[68]
miR-105	Intracellular	Triple negative breast cancer tissue	↑	WNT/β-catenin	Promotion of metastasis, stemness and chemoresistance	[73]
miR-126	Extracellular	Blood sample	↓	-	Good metastatic colorectal cancer therapy response (chemotherapy and bevacizumab combination)	[88]
miR-128b	Extracellular	Peripheral blood, bone marrow	↑	PTEN	Good leukemia therapy response (prednisolone)	[89]

↑ Upregulation; ↓ downregulation; PIK3/AKT: phosphatidylinositol-4,5-bisphosphate 3-kinase/AKT serine-threonine kinase; PTEN: phosphatase and tensin homolog; “-”: no described target.

**Table 3:** Examples of miRNAs as cancer follow-up biomarkers.

ECmiRNAs	Level	Sample	Cancer	Effect	Ref.
miR-128, miR-139-5p, miR-382-5p, miR-410	↑	Blood	Hepatocellular carcinoma	Larger tumor size, cancer invasion and worst prognosis	[107]
miR-29c, miR-143-3p, miR-187-3p, miR-205-5p, miR-210	↓	Blood	Breast cancer	Good prognosis, up to 10-year life expectancy	[108]
miR-940, miR-451a, miR-16-5p, miR-17-3p	↑	Serum	ERBB2+ metastatic breast cancer	Trastuzumab treatment response and good cancer prognosis	[109]
miR-20a, miR-222, miR-20b, miR-132, miR-25	=	Serum	Prostate cancer	Docetaxel resistance and poor cancer prognosis	[110]
miR-1	↑	Plasma	Breast cancer	Doxorubicin-induced cardiotoxicity	[118,119]
miR-126-3p, miR-199a-3p, miR-423-5p, miR-34a-5p	↑	Plasma	Breast cancer	Congestive heart failure as consequence of anthracycline cancer treatment	[120]
miR-194, miR-146-3p	↑	Serum	Prostate cancer	Cancer recurrence	[112]
miR-17, miR-20a, miR-20b, miR-106a	↑	Blood	Prostate cancer	Cancer recurrence	[113]
miR-1225-3p, miR-1234-3p, miR1260b, miR-1470	↑	Serum	Biliary tract cancer	Cancer recurrence; poor prognosis	[114]
miR-6834-3p, miR-6875-5p	↓				

↑ Upregulation; ↓ downregulation; = unchanged; ERBB2: erb-b2 receptor tyrosine kinase 2.

## Data Availability

Not applicable.
